# Association Between Genetic Variants in *DEFB1* Gene and Dental Caries Susceptibility

**DOI:** 10.1155/ijod/8465309

**Published:** 2025-10-28

**Authors:** Mohammad Javad Mokhtari, Fatemeh Koohpeima, Farzad Hashemi-Gorji, Reza Mirfakhraie, Vahid Reza Yassaee, Majid Marjani, Mohammad Varahram, Mostafa Rezaei-Tavirani, Navid Omidifar, Mitra Rezaei

**Affiliations:** ^1^Department of Biology, Zarg. C., Islamic Azad University, Zarghan, Iran; ^2^Department of Oral Medicine, School of Dentistry, Shiraz University of Medical Sciences, Shiraz, Iran; ^3^Genomic Research Center, Shahid Beheshti University of Medical Sciences, Tehran, Iran; ^4^Clinical Tuberculosis and Epidemiology Research Centre, National Research Institute for Tuberculosis and Lung Disease, Shahid Beheshti University of Medical Sciences, Tehran, Iran; ^5^Proteomics Research Center, System Biology Institute, Faculty of Paramedical Sciences, Shahid Beheshti University of Medical Sciences, Tehran, Iran; ^6^Department of Pathology, School of Medicine, Shiraz University of Medical Sciences, Shiraz, Iran

**Keywords:** *DEFB1*, dental caries, polymorphism, tetra-primer ARMS–PCR

## Abstract

**Introduction:**

Genetics plays a crucial role in determining individual susceptibility to dental caries. Certain *beta-defensin 1* (*DEFB1*) gene variants may be linked to oral diseases. One of the most prevalent problems with oral health is dental caries. Between 60% and 90% of schoolchildren and the vast majority of adults suffer from dental caries. This study aimed to examine the genetic association between the *DEFB1* gene variants rs11362 and rs1799946 and dental caries in a case–control study.

**Methods:**

The study included 202 participants, of whom 119 demonstrated high dental caries, while 83 acted as controls with low dental caries. Most of them were referred to the Medical University's Dental Clinic in Shiraz, Iran. Following the acquisition of written and informed consent, genomic DNA was extracted from peripheral blood. Tetra-primer ARMS–PCR was employed to identify the *DEFB1* promoter region polymorphisms rs11362 and rs1799946.

**Results:**

Allelic, genotypic, and haplotypes frequencies did not differ between the case and control groups, according to the genotyping results.

**Conclusion:**

To find the dental caries-vulnerable loci in our case and control groups, more studies with a bigger sample size are essential. Furthermore, functional research is necessary to elucidate how these variations affect *DEFB1* expression.

## 1. Introduction

Caries impact the majority of humans, despite a demonstrated decrease in its prevalence across all age demographics [1]. The host defense mechanisms, the structure of individual teeth, and the composition of saliva and pellicle influence the severity of caries and may complicate disease prevention [2]. Genetics accounts for 20%–60% of caries in individuals, with a more pronounced influence observed in deciduous teeth compared to permanent teeth [3].

As a member of the defensin family, human *beta-defensin 1 (DEFB1)* is a 36-amino acid antimicrobial peptide [4]. The *DEFB1* gene encodes this peptide, which is the main defense against dental caries and is found in the mucosal surfaces of the gastrointestinal tract, urogenital tissue, salivary glands, and oral tissue [4–6]. The *DEFB1* gene is linked to immune response, functioning as a host defense protein that impacts both the innate and adaptive immune systems, thereby influencing the progression of dental caries [7, 8].

Gene polymorphisms can affect the function and expression level of genes that lead to human disorders. Single nucleotide polymorphism (SNP) is caused by point mutations that affect only one nucleotide within a genetic sequence that gives rise to different alleles [9]. Numerous investigations have been carried out concerning the relationship between polymorphisms of genes involved in immunological responses and dental caries [8, 10]. The relationship between *DEFB1* gene variants and dental caries in the Iranian population has not yet been investigated. Prior research examined the correlation between caries prevalence and variants in *DEFB1* [11–14]. Yildiz et al. [11] demonstrated that allele A (rs11362) is more prevalent in individuals with a high risk of caries. A separate study observed that the CC genotype (rs11362) is associated with increased susceptibility to caries [12]. Ozturk et al. [13] indicate that *DEFB1* contains significant polymorphic variants associated with either an increased (rs11362) or decreased (rs1799946) incidence of caries, which may serve as clinical markers for caries risk in humans. Additionally, the GCA haplotype was identified, which doubled decayed, missing, and filled teeth (DMFT) scores, while the ACG haplotype was found to halve DMFT scores, both located in the *DEFB1* promoter [13]. Other research indicated that polymorphisms in *DEFB1* were not correlated with caries experience [14]. Additionally, two meta-analyses indicated a correlation between *DEFB1* variants and the risk of dental caries [4].

This study examined *DEFB1* SNPs (rs11362 and rs1800972) in populations with high and low dental caries to ascertain the relationship between *DEFB1* genetic polymorphisms and dental caries risk in the Iranian demographic. This is the first report examining the association of *DEFB1* SNPs with dental caries risk in the Iranian population. Our objective was to determine if *DEFB1* polymorphisms influence the early pathogenesis of disease and to assess the potential clinical significance of genetic testing for *DEFB1* in the early detection and proactive management of dental caries.

## 2. Materials and Methods

### 2.1. Study Subjects

All procedures in this study were approved by the Deputy of Research Affairs' Ethical Committee at Shahid Beheshti University of Medical Sciences in Tehran, Iran, under approval ID: IR.SBMU.RETECH.REC.1403.065, in compliance with the 1964 Helsinki Declaration and its subsequent amendments or equivalent ethical standards. The study was conducted on both male and female participants who were referred to the Medical University of Shiraz, Iran. Every individual had a dental examination conducted by a dentist. The individuals' written and informed consent was obtained before this was done. Standard dental chairs, artificial lighting, and a flat mirror and explorer were used for clinical dental examinations. Bitewing and specific periapical radiographs were subsequently obtained. The number of DMFT was used to calculate the incidence of caries. The G-Power software package was used to perform a power analysis. A minimum of 70 patients was determined to be requisite for both the low and high dental caries risk groups, with *α* = 0.05 for type I error and *β* = 0.05 for type II error rates, attaining 90% power with an effect size of *d* = 0.40. A total of 202 individuals were chosen: 83 for the low dental caries risk category and 119 for the high dental caries risk category. The cases were defined as individuals diagnosed with high caries (DMFT > 6), while controls were defined as individuals with lower caries and no history of caries [10]. The criteria for inclusion and exclusion are detailed in Table 1. In the present study, brushing and flossing frequency were examined as indicators of dental hygiene, while education level was analyzed as a key indicator of socioeconomic status. Among both case and control groups, over 90% had a brushing frequency of once per day and flossing once per day or less. No significant differences were detected (*p* > 0.05). Also, no statistically significant difference was observed in education level between case and control groups (*p* > 0.05). Concerning the case group exhibited a marginally higher daily sugar intake and frequency of consumption compared to the control group, the differences did not reach statistical significance. Regarding periodontal disease, subjects with clinical signs of severe periodontal disease (e.g., generalized probing depths ≥ 6 mm, bleeding on probing, or radiographic bone loss > 30%) were excluded based on prior dental records or clinical examination conducted at baseline.

### 2.2. DNA Extraction and Genotyping

Blood samples were collected and stored at − 20°C in EDTA-containing tubes. Genomic DNA for genotyping was extracted using the salting out method [15]. Genotyping of two variants in the promoter-regulatory region of *DEFB1* (rs11362 [-20G>A] and rs1799946 [-52G>A]) was conducted using the tetra-primer ARMS technique with specific primers. Two sets of primers were used in a single vial for a single PCR reaction [16]. A 2.5% agarose gel was used to validate the PCR products to ensure the accuracy of the genotyping results.  Table 2 displays the primer sequences and the parameters for the PCR reaction.

### 2.3. Statistical Analysis

The chi-squared (χ^2^) test was used to compare the expected and observed genotype frequencies in the control group to evaluate the Hardy–Weinberg equilibrium (HWE). Means ± standard deviation (SD) is used to report mean ages. To evaluate different genetic models, unconditional logistic regression analysis was employed. Included are the 95% confidence interval (CI) and the odds ratio (OR). *p* < 0.05 was used to determine that the results were statistically significant. SPSS software version 19.0 was used to conduct the analyses.

## 3. Results

### 3.1. General Characteristics of the Studied Population

The study group consisted of 119 participants with high dental caries, including 74 females (62.18%) and 45 males (37.81%), aged 18–58, with a mean age of 29.45 and a SD of 8.09. The control group comprised 83 individuals (40 females: 48.19% and 43 males: 51.80%) with low dental caries, aged between 17 and 65 years (28.72 ± 9.56).

### 3.2. Genotyping of *DEFB1* rs11362 and rs1799946 Polymorphisms

**Figure 1:**
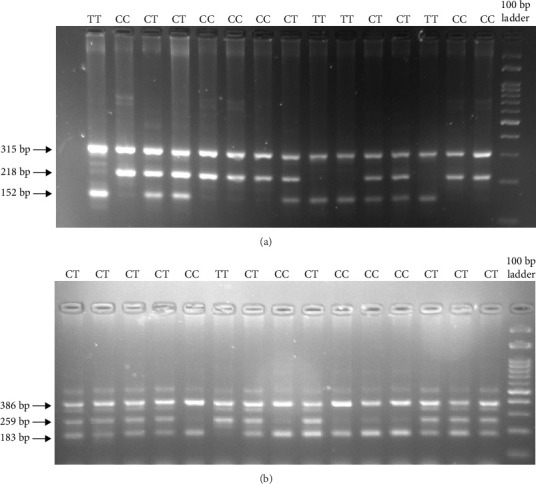
(a) Tetra-primer ARMS-PCR to identify the genotype of *DEFB1* rs11362 genotype. The product sizes were 152 bp for the T allele, 218 bp for the C allele, and 315 bp for internal control. (b) Tetra-primer ARMS-PCR for the detection of DEFB1 rs1799946 genotype. The product sizes were 183 bp for the C allele, 259 bp for the T allele, and 386 bp for internal control.

The genotyping of *DEFB1* rs11362 and rs1799946 was conducted utilizing the tetra-primer ARMS-PCR technique, as shown in Figure 1. The analysis of the genotypes of the rs11362 variant revealed that the frequencies of TT, CT, and CC genotypes in the high dental caries group were 20 (16.80%), 56 (47.05%), and 43 (36.16%), while in the low dental caries group, they were 14 (16.86%), 40 (48.16%), and 29 (34.93%), respectively. The genotype distribution indicated that both the low dental caries group (χ^2^ = 0.001, df = 1, and *p*=0.97) and the high dental caries group (χ^2^ = 0.059, df = 1, and *p*=0.80) are in HWE. For rs1799946, the frequencies of the TT, CT, and CC genotypes in the high dental caries group were 30 (25.21%), 52 (43.69%), and 37 (31.09%), respectively, while in the low dental caries group, they were 26 (31.32%), 32 (38.55%), and 25 (30.12%). The genotype distribution demonstrated that the high dental caries groups adhered to HWE (χ^2^ = 1.80, df = 1, and *p*=0.17).

### 3.3. rs11362 and rs1799946 Variants and Dental Caries Risk

We observed that the TT and CT genotypes were less common and the CC genotype was more common in the high dental caries group relative to the control group for SNP rs11362; the OR was determined to be nonsignificant (*p* > 0.05). There was no significant difference in the risk of dental caries between the T and C alleles (OR = 1.02, 95% CI: 0.68–1.53, and *p*=0.89) (Table 3). Additionally, for rs1799946, we observed a greater prevalence of the CC and CT genotypes and a reduced prevalence of the TT genotype in the high dental caries group compared to the low dental caries group. There was no significant difference in the risk of dental caries between the C and T alleles (OR = 0.86, 95% CI: 0.58–1.29, and *p*=0.48) (Table 3).

### 3.4. Distribution of Haplotypes Among High Dental Caries Group and Low Dental Caries Group

The frequency distribution of haplotypes is shown in Table 4. In the four haplotype models (rs11362 and rs1799946), haplotypes CT and TC exhibited a higher frequency in the high dental caries group relative to the low dental caries group, while haplotypes CC and TT demonstrated a lower frequency in the high dental caries group compared to the low dental caries group; the OR was determined to be nonsignificant.

## 4. Discussion

This research aimed to ascertain the correlation between *DEFB1* SNPs rs11362 and rs1799946 and the susceptibility to dental caries in a southwestern Iranian population. There was no significant difference between the rs11362 and rs1799946 polymorphisms and dental caries. The OR values were approximately neutral at “1”. Consequently, they demonstrated that the results lacked statistical significance. Moreover, haplotype analysis indicated a nonsignificant association between SNPs and dental caries. This study represents the inaugural examination of the correlation between *DEFB1* SNPs rs11362 and rs1799946 polymorphisms and the risk of dental caries in the Iranian population.

Research studies indicate that genetic factors significantly influence the development of dental caries, focusing on four primary gene groups related to tooth enamel formation, saliva composition, immune response, and carbohydrate metabolism [8]. Numerous studies have examined the correlation between SNPs in genes that encode antimicrobial salivary peptides and the susceptibility to dental caries within the realm of genes influencing immune responses. Azevedo et al. [17] observed a correlation between allele A of *lactoferrin* (exon 2 *LTF*) and reduced caries intensity, along with enhanced salivary flow, suggesting its protective function. Abbasoğlu and associates also found a link between a variation of the *LTF* gene and decreased caries susceptibility, proving that the CT genotype protects against caries in early childhood [18]. Fine et al. [19] obtained noteworthy results by identifying an *LTF* gene variant associated with lactoferrin activity in saliva, enhancing the antimicrobial response. This variant leads to reduced caries intensity in affected patients [19]. The prior study identified a correlation between polymorphism in the *MBL2* gene and dental caries in Iranian adults. The combined estimates showed that the *MBL2* rs11003125 polymorphism's GG and GC genotypes markedly increased caries risk [10]. Olszowski et al. [20] studied the polymorphisms of *MBL2*. In the 5-year-old cohort with elevated caries, they found that the G allele (rs11003125) was more prevalent than in the cohort with minimal dental caries [20]. Alyousef et al. [21] demonstrated that the rs11003125 G allele was correlated with dental caries.

Numerous studies [22, 23] have assessed the significance of antimicrobial peptides in innate immunity and oral health. These molecules exhibit considerable efficacy against bacteria, serving as a primary defense against diverse pathogens. Human *DEFB1s*, a significant class of antimicrobial peptides, have exhibited in vitro effectiveness against oral microorganisms [14]. *DEFB1* is an oral antimicrobial peptide that functions as the principal defense against various pathogens [4]. Defensin levels in saliva and oral tissues vary significantly, which can be attributed to host genetic variations [24]. Prior research indicated that polymorphisms in the *DEFB1* promoter region modify transcriptional activity relative to the wild-type region [25, 26]. This variation may correlate with differences in caries experience among individuals. Ozturk et al. showed that the DMFT and DMFS scores increased more than five times when a variant allele of the *DEFB1* marker rs11362 was present. The GCA haplotype that doubled DMFT scores and the ACG haplotype ACG that halved DMFT scores are situated in the *DEFB1* promoter [13]. Recent systematic reviews and meta-analyses underscore the significance of SNPs in influencing caries susceptibility in children [4, 8]. Studies have shown that *DEFB1* variants may affect antimicrobial peptide expression in saliva, thereby altering the host's defense mechanism against cariogenic pathogens [13]. Furthermore, genetic variants involved in enamel formation and immune response pathways have demonstrated associations with early childhood caries [18], while regulatory interactions involving microRNA202 have also emerged as a potential modulating factor [27]. Nevertheless, contradictory findings exist, including studies that found no association between *DEFB1* polymorphisms and caries in primary teeth [28]. Such discrepancies highlight the multifactorial nature of caries etiology and suggest that genetic predisposition may vary across populations. In this context, our study contributes to the evolving understanding of gene–environment interactions in caries development and reinforces the need for integrative genomic approaches in oral health research.

Two polymorphisms in the promoter region (rs11362 and rs1799946) are linked to caries experience, according to previous studies that evaluated the relationship between caries experience and polymorphisms in *DEFB1* [11–13]. Our study's multivariate analysis did not reveal a correlation between the rs11362 and rs4547741 variants and the caries experience. This disparity may be attributable to the heterogeneity within the examined populations. Prior studies were conducted on North American adults [13], Latvian children with oral clefts [12], and Turkish adults [11], whereas our research focused on the Iranian population, which is notably heterogeneous, for genetic analysis [29]. Indeed, the research conducted by Lips et al. yielded results analogous to those of our study. The polymorphisms in *DEFB1* were found to be unassociated with caries experience [14].

This study is the first investigation into the association between *DEFB1* variants rs11362 and rs1799946 with dental caries risk in the Iranian population. Although this study did not find statistically significant associations between *DEFB1* polymorphisms and dental caries, it contributes valuable baseline data for future research. Null findings are essential to balance potential publication bias and provide a comprehensive understanding of genetic influence on caries. Our findings differ from previous studies, possibly due to population heterogeneity, environmental exposures, or gene–environment interactions unique to the Iranian population. This highlights the importance of regional studies in genetic epidemiology. Also, the allele and genotype frequency data provided herein may serve as a useful reference for future meta-analyses aimed at consolidating *DEFB1*-related genetic findings across diverse populations. Our research exhibited multiple limitations. Information on each person's diet, dental health, bacterial infections in the mouth, and fluoride exposure was unavailable. These factors are recognized as modifying determinants for caries. The limited sample size may have influenced the results. Furthermore, our study did not investigate the impact of *DEFB1* variants on its expression and functionality. Hence, we strongly advise conducting similar investigations on a larger sample size to validate whether *DEFB1* SNPs, specifically rs11362 and rs1799946, indeed impact the expressions of this gene and contribute to the susceptibility of dental caries.

## 5. Conclusion

This study is the first to investigate the possible association between dental caries in Iranian adults and *DEFB1* variants. This research is the inaugural report examining the potential association between *DEFB1* polymorphisms and dental caries in Iranian adults. This research revealed no significant correlation between the rs11362 and rs1799946 variants and an elevated risk of dental caries. Furthermore, none of the haplotypes (rs11362 and rs1799946) in the diverse haplotype models demonstrated a correlation with the risk of dental caries. Nonetheless, given that various factors can significantly influence the pathogenesis of dental caries, it would be prudent to undertake additional genetic studies across diverse ethnic populations with an expanded sample size.

## Figures and Tables

**Table 1:** Patient inclusion and exclusion criteria.

Criteria	Description
Inclusion	(i) The patients, both male and female, ranged in age from 17 to 65 (ii) All subjects' socioeconomic status and dental hygiene practices were similar (iii) The participants willingly put their signatures on a consent form, which was endorsed by the ethics committee of Shiraz University of Medical Sciences, located in Shiraz, Iran

Exclusion	(i) History of smoking and alcohol consumption (ii) The presence of any systemic diseases (iii) History of traumatic injury-related tooth loss (iv) Severe periodontal disease coexisting (v) Pregnant women (vi) presence of serious mental disorders

**Table 2:** Primer sequences and PCR conditions related to the amplification of *DEFB1* polymorphisms.

SNP	Primer name	Primer sequence 5′-3′	Length	PCR condition	PCR product size
rs11362 C > T	rs11FI	AGTTCTCATGGCGACTGGCAGGCAACCCT	29	95:3 min 95:35 S 64.5:35 S 72:35 S 35 cycles	152 bp (T allele)
	rs11RI	AGCGTCTCCCCAGTTCCTGAAATCCGGG	28		218 bp (C allele)
	rs11FO	GCTGCTTGTTCCTCGTCCCTTGGGACAC	28		Internal Control 315 bp
	rs11RO	CAGTTCCGTCGACGAGGTTGTGCAATCC	28		

rs1799946 C > T	rs17FI	GATTTCAGGAACTGGGGAGACGCTGGATC	29	95:3 min 95:40 S 55:40 S 72:40 S 30 cycles	183 bp (C allele)
	rs17RI	AGCCTCTGTCAGCTCAGCCTCCAACGA	27		259 bp (T allele)
	rs17FO	ATGCTTTCCTGCTGCTTGTTCCTCGTCC	28		Internal Control 386 bp
	rs17RO	AGGGCAAATGACACCAGGGGTTAGCGAT	28		

**Table 3:** Distribution of genotypes and allele frequencies of *DEFB1* (rs11362 and rs1799946) gene polymorphisms in the cases and control groups in different genetic models.

SNP	Genotype	Case group (%)	Control group (%)	OR (95% CI)	*p*-Value
rs11362 Co-dominant	TT CT CC	20 (16.80) 56 (47.05) 43 (36.16%)	14 (16.86) 40 (48.19) 29 (34.93)	1 0.98 (0.44–2.16) 1.03 (0.45–2.37)	0.96 0.92

Dominant	TT	20 (16.80)	14 (16.86)	1	—
	CT + CC	99 (83.19)	69 (83.13)	1.00 (0.47–2.12)	0.99

Recessive	CT + TT	76 (63.86)	54 (65.06)	1	—
	CC	43 (36.16%)	29 (34.93)	1.05 (0.58–1.89)	0.86

Over-dominant	CC + TT	63 (52.94)	43 (51.80)	1	—
	CT	56 (47.05)	40 (48.19)	0.95 (0.54–1.67)	0.87

—	T allele C allele	96 (40.33) 142 (59.66)	68 (40.96) 98 (59.03)	1 1.02 (0.68–1.53)	0.89

rs1799946 Co-dominant	TT CT CC	30 (25.21) 52 (43.69) 37 (31.09)	26 (31.32) 32 (38.55) 25 (30.12)	1 1.40 (0.70–2.79) 1.28 (0.61–2.66)	0.32 0.50

Dominant	TT	30 (25.21)	26 (31.32)	1	—
	CT + CC	89 (74.78)	57 (47.89)	1.35 (0.72–2.51)	0.33

Recessive	CT + TT	82 (68.90)	58 (69.87)	1	—
	CC	37 (31.09)	25 (30.12)	1.04 (0.56–1.92)	0.88

Over-dominant	CC + TT	67 (56.30)	51 (61.44)	1	—
	CT	52 (43.69)	32 (38.55)	1.23 (0.69–2.19)	0.46

—	C allele T allele	126 (52.94) 112 (47.05)	82 (49.39) 84 (50.60)	1 0.86 (0.58–1.29)	0.48

**Table 4:** Analysis of the haplotype frequencies of *DEFB1* between cases and controls.

Haplotype		Case (frequency)	Control (frequency)	*p*-Value	OR (95% CI)
rs11362	rs1799946				
C	T	0.399	0.390	—	1
T	C	0.332	0.294	0.78	1.07 (0.68–1.68)
C	C	0.197	0.199	0.74	0.90 (0.50–1.64)
T	T	0.071	0.115	0.17	0.54 (0.22–1.31)

## Data Availability

The primer sequences for PCR are provided in Table 2. All data generated or analyzed during this study are included in this article. Further enquiries can be directed to the corresponding author.
